# Supplementary material for: Multi-view clustering by CPS-merge analysis with application to multimodal single-cell data
Source: PLoS Comput Biol. 2023 Apr 17;19(4):e1011044. doi: 10.1371/journal.pcbi.1011044 (PMC10138214; doi:10.1371/journal.pcbi.1011044)
Supplement: S1 Appendix — (PDF) [file pcbi.1011044.s001.pdf]

S1 Appendix

1 Simulation Study

We investigate three simulation cases in which complementary information exists between views, *e.g.*, no dominating views for any cluster, some views are dominating for clustering, and clustering with more than 2 views. The summary of the 3 datasets is given in Supplementary Table A. The simulation study is motivated by our observation that in multimodal scRNA-deq data, different views often give complementary information about the overall clustering result, and clusters in different views demonstrate little consensus. Intuitively, existing ensemble clustering methods, *e.g.*, hybrid bipartite graph formulation [1], are likely to fail because they rely totally on the consensus principle. Moreover, when clusters are formed based on complementary information across views, assessing the cluster-wise contribution of each view is of great interest. For example, in some single-cell data, certain types of T cells are not well-separated according to the transcriptome alone. By measuring the contribution of each view to the formation of a cluster, we gain an understanding of how much another modality helps the transcriptome. This measure of cluster-wise contribution is not available in existing ensemble clustering methods or multi-view clustering methods. In Supplementary Table B, accuracy by the WNN method is not reported for the simulation datasets because this method, with a pipeline specifically designed for multimodal single-cell data, failed to yield meaningful results. Accuracy by the Co-train or WNN method is not reported for data with 3 views (Simulation data 3) because these methods only handle 2 views. The results show that CPS-merge analysis is not only able to handle the complementary information about clustering, but provides meaningful cluster-wise contribution as well.

S1 Appendix Table A. Summary of the 3 multi-view simulation datasets.

Dataset	Samples	View Dimensions	# Clusters
Sim1	1000	(10, 10)	4
Sim2	1000	(10, 10)	4
Sim3	1000	(10, 10, 10)	4

S1 Appendix Table B. Clustering results on 3 muti-view simulation datasets (rows) by 7 methods (first 7 columns) in terms of ARI, NMI and F-measure. Columns from View1 to View3 are single-view clustering results on each dataset. The highest ARI, NMI and F-measure for each dataset are in bold.

ARI<br>(NMI)<br>[F-measure]	Co-train	MKC	MSC	Ensemble	DeepCC	CCA	CPS-merge	A-CPS-merge	View 1	View 2	View 3
Sim1	0.300<br>(0.277)<br>[0.535]	0.992<br>(0.987)<br>[0.994]	0.335<br>(0.488)<br>[0.503]	0.349<br>(0.510)<br>[0.513]	0.623<br>(0.637)<br>[0.734]	**1**<br>**(1)**<br>**[1]**	**1**<br>**(1)**<br>**[1]**	**1**<br>**(1)**<br>**[1]**	0.335<br>(0.501)<br>[0.502]	0.333<br>(0.500)<br>[0.501]	
Sim2	0.713<br>(0.710)<br>[0.796]	**1**<br>**(1)**<br>**[1]**	0.702<br>(0.738)<br>[0.777]	0.954<br>(0.951)<br>[0.966]	0.714<br>(0.765)<br>[0.795]	0.665<br>(0.749)<br>[0.764]	**1**<br>**(1)**<br>**[1]**	**1**<br>**(1)**<br>**[1]**	0.668<br>(0.749)<br>[0.752]	0.725<br>(0.772)<br>[0.795]	
Sim3		0.997<br>(0.995)<br>[0.998]	0.374<br>(0.404)<br>[0.536]	0.826<br>(0.819)<br>[0.869]	0.600<br>(0.659)<br>[0.715]	0.628<br>(0.748)<br>[0.736]	**1**<br>**(1)**<br>**[1]**	**1**<br>**(1)**<br>**[1]**	0.336<br>(0.410)<br>[0.502]	0.326<br>(0.404)<br>[0.494]	0.309<br>(0.396)<br>[0.482]

1.1 Case 1: No dominating views for any cluster

We generate a sample of 1000 data points with 20 variables from a 4-component Gaussian mixture model. In particular, we assign the same prior probability to the 4 components. The mean vectors for the 4 components are $(5 \cdot \mathbf{1}_{10}^T, 10 \cdot \mathbf{1}_{10}^T), (5 \cdot \mathbf{1}_{10}^T, 5 \cdot \mathbf{1}_{10}^T), (10 \cdot \mathbf{1}_{10}^T, 10 \cdot \mathbf{1}_{10}^T), (10 \cdot \mathbf{1}_{10}^T, 5 \cdot \mathbf{1}_{10}^T)$, respectively. The covariance matrices are all set to be diagonal with diagonal elements equal to 1. Each component is treated as one cluster, while we regard the first 10-dimensional variables as features observed from the first view and the last 10-dimensional variables as features observed from the second view. In this setup, there is no consensus on clustering between the two views, and neither of the views can fully discern any final cluster. Not surprisingly, the ensemble clustering method with the underlying consensus principle yields poor results. In contrast, CPS-merge analysis exploits complementary information when aggregating clustering results.

For each view, we set the number of clusters k to 4. We also carried out a robustness analysis, letting k vary from 3 to 6. At each k, CPS-merge analysis and its accelerated version both converge to the true clustering result (i.e., the true mixture component), yielding ARI equal to 1. We then applied Kmeans to both the original data and the collection of perturbed data to obtain the corresponding clustering results. The single-view clustering and CCA (concatenation cluster analysis) method in Supplementary Table B also use Kmeans as the clustering method. The true components and the clustering result in each view are visualized in Supplementary Fig A by the UMAP visualization method. In view 1, true components 1 and 2 are separated from components 3 and 4; and in view 2, true components 1 and 3 are separated from components 2 and 4. But none of the clusters identified in view 1 or view 2 alone is a true component. We then applied the CPS-merge analysis. The analysis yields a result with every cluster bearing a tightness value above 0.98. As shown in Supplementary Table B, the final clustering result by the CPS-merge analysis achieves an ARI equal to 1 (identical to truth). But all the competing methods except for MKC yield low ARI. CCA method works in this case that the information from both views is fully complementary. MKC performs similarly to CPS-merge analysis, but does not evaluate the cluster-wise contribution of each view.

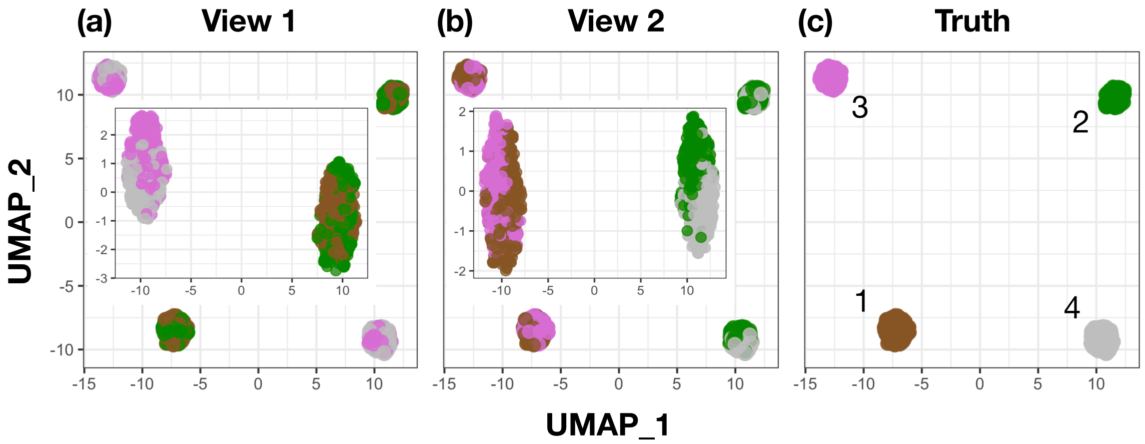

S1 Appendix Fig A. UMAP visualization of the first simulated dataset and the clustering results. (a) Single-view clustering result in View 1. (b) Single-view clustering result in View 2. (c) True clusters. The large-frame plots in (a) to (c) are based on UMAP visualization of the entire data (combining both views). The two embedded mini-frame plots in (a) and (b) are based on UMAP visualization of the corresponding single-view data. Clusters are indicated by different colors.

According to CPS-merge analysis both the tightness-based contribution and the matching-weight-based contribution of each view are close to 0.5. Specifically, the tightness of each cluster in the first view is respectively 0.208, 0.218, 0.208, 0.202, and the tightness of each cluster in the second view is 0.182, 0.198, 0.194, 0.174. The low tightness values of all the clusters in individual views indicate that clusters in the final result are formed based on complementary information from both views.

1.2 Case 2: Some views are dominating for clustering

Similar to Case 1 in Section 1.1, we generate a sample of 1000 data points with 20 variables from a 4-component Gaussian mixture model. The difference lies in the mean vectors. In Case 2, the mean vectors for the 4 components are $(5 \cdot \mathbf{1}_{10}^T, 5 \cdot \mathbf{1}_{10}^T), (15 \cdot \mathbf{1}_{10}^T, 10 \cdot \mathbf{1}_{10}^T), (10 \cdot \mathbf{1}_{10}^T, 10 \cdot \mathbf{1}_{10}^T), (10 \cdot \mathbf{1}_{10}^T, 15 \cdot \mathbf{1}_{10}^T)$, respectively. Again, we take the first 10-dimensional variables as features in the first view and the last 10-dimensional variables as features in the second view. As will be elaborated on below, some clusters found in individual views are in consensus, while others reveal complementary patterns.

There is one component identified as a cluster in both views, and the other components can be distinguished by either complementary information or strong evidence in a single view. More specifically, in both views, component 1 is extracted as one cluster. Component 2 can be distinguished in view 1, and component 4 can be distinguished in view 2. To identify component 3, we need combined information from both views. We then carry out CPS-merge analysis, and the algorithm converges to a result containing clusters each with tightness above 0.93. The clustering results are shown in Supplementary Fig B. From Supplementary Table B, we see that the final clustering result by CPS-merge analysis achieves ARI equal to 1. In contrast, the other competing methods except for MKC cannot identify every component. In particular, CCA achieves roughly the same level of performance as sing-view clustering when the information is not fully complementary.

We compute the tightness-based contribution of each view for every cluster in the final clustering result. The contribution of view 1 for the four clusters respectively is $(0.486, 0.786, 0.559, 0.324)$, while the contribution

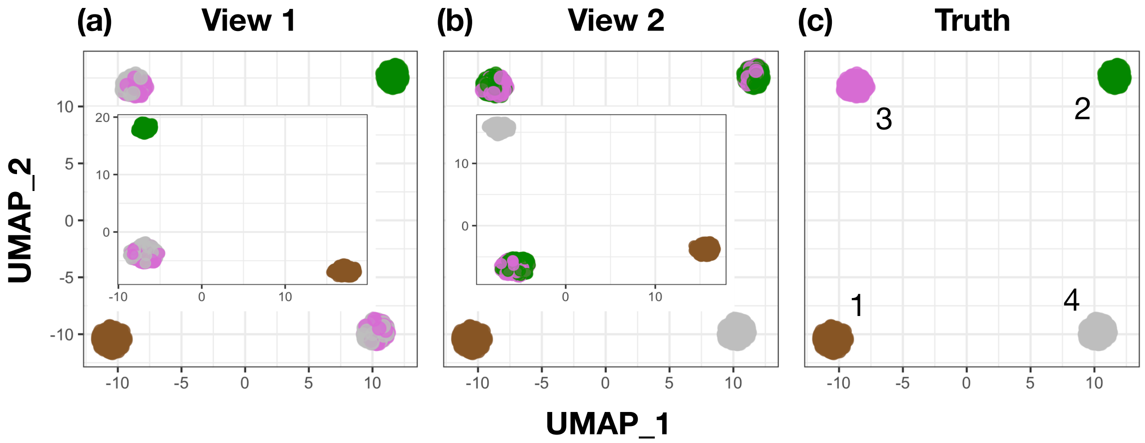

S1 Appendix Fig B. UMAP visualization of the second simulated dataset and the clustering results.
(a) Single-view clustering result in View 1. (b) Single-view clustering result in View 2. (c) True clusters. The large-frame plots in (a) to (c) are based on UMAP visualization of the entire data (combining both views). The two embedded mini-frame plots in (a) and (b) are based on UMAP visualization of the corresponding single-view data. Clusters are indicated by different colors.

of view 2 is $(0.514, 0.214, 0.441, 0.676)$. Note that the contribution of view 1 to component 2 and that of view 2 to component 4 are both high, concurring with the truth. Also, both views have similar contribution values to components 1 and 3, but the views play different roles for the two components. For component 1, both views yield high tightness (0.710 and 0.750), suggesting that this component exists in consensus. For component 3, both views yield low tightness (0.294 and 0.232), suggesting that this component is formed by complementary information.

1.3 Case 3: More than two views

We use this example to demonstrate that CPS-merge analysis can handle datasets with more than two views. Again a sample of 1000 points with 30 variables is generated by a 4-component Gaussian mixture model. The mean vectors for the 4 components are $(5 \cdot \mathbf{1}_{10}^T, 10 \cdot \mathbf{1}_{10}^T, 10 \cdot \mathbf{1}_{10}^T), (10 \cdot \mathbf{1}_{10}^T, 5 \cdot \mathbf{1}_{10}^T, 10 \cdot \mathbf{1}_{10}^T), (10 \cdot \mathbf{1}_{10}^T, 10 \cdot \mathbf{1}_{10}^T, 5 \cdot \mathbf{1}_{10}^T), (10 \cdot \mathbf{1}_{10}^T, 10 \cdot \mathbf{1}_{10}^T, 10 \cdot \mathbf{1}_{10}^T)$. The first, second, and third views contain respectively the first, second, and third blocks of 10-dimensional variables.

In the design of this simulation, we let the ith view provide the essential information for component i, $i = 1, 2, 3$ but the 4th component requires all the 3 views to specify. The true components and the clustering results are shown in Supplementary Fig C. The CPS-merge analysis analysis converges to a clustering result containing clusters all with tightness above 0.99. Supplementary Table B shows that the CPS-merge analysis achieves ARI equal to 1 again, while other methods fail to discover every component.

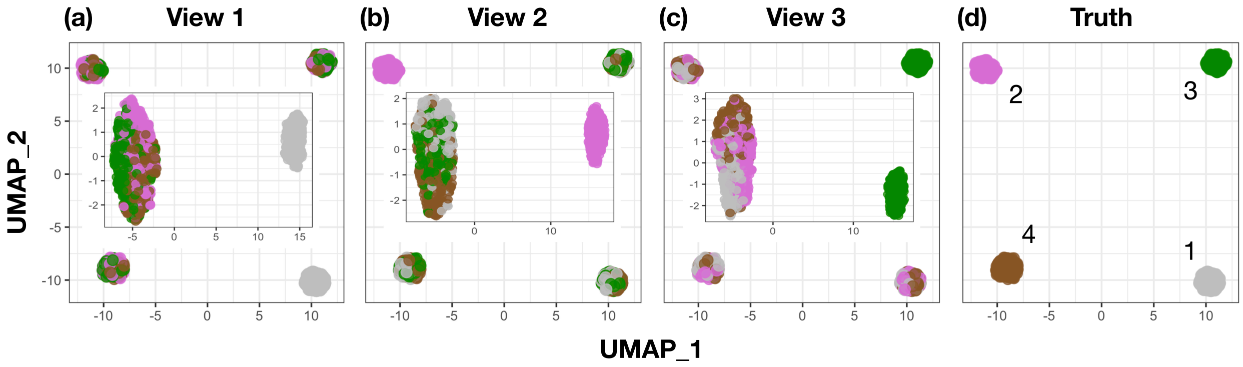

S1 Appendix Fig C. UMAP visualization of the third simulated dataset and the clustering results.
(a) Single-view clustering result in View 1. (b) Single-view clustering result in View 2. (c) Single-view clustering result in View 3. (d) True clusters. The large-frame plots in (a) to (d) are based on UMAP visualization of the entire data (combining both views). The three embedded mini-frame plots in (a) to (c) are based on UMAP visualization of the corresponding single-view data. Clusters are indicated by different colors.

The tightness-based contribution of view i for component i, $i = 1, 2, 3$, is respectively 0.598, 0.569, and 0.645. In contrast, the contribution of every view for component 4 is around 0.33, and the tightness of this component in each view is around 0.25, indicating that component 4 is formed based on complementary information in all the views. These findings on the cluster-wise contribution of views are consistent with how the simulation datasets are generated.

2 Sensitivity Analysis

S1 Appendix Table C. Sensitivity analysis of number of final clusters in CPS-merge analysis using 3 multimodal single-cell datasets. We report the average performance (Mean) and standard deviation (sd) in terms of ARI, NMI and F-measure.

	ARI	NMI	F
Dataset	Mean (sd)	Mean (sd)	Mean (sd)
HBMC	0.813 (0.017)	0.814 (0.003)	0.832 (0.015)
PBMC1	0.788 (0.032)	0.809 (0.017)	0.813 (0.029)
PBMC2	0.729 (0.085)	0.796 (0.017)	0.761 (0.078)

We first analyze the robustness of CPS-merge analysis when the number of final clusters, denoted by κ_1, is not certain. Using the multimodal single-cell datasets as examples, we evaluate the performance of CPS-merge when κ_1 deviates from the true value by up to 3 (either smaller or larger), that is, 7 different values in total. In Supplementary Table C, we report the average clustering performance (in terms of ARI, NMI and F-measure) and the standard deviation across the 7 different values of κ_1. Compared with the results shown in Supplementary Table B, the average ARIs of CPS-merge under different κ_1 remain higher than the ARI by any other competing method using the true number of clusters. The low standard deviations reflect the fact that the performance is stable when κ_1 varies. The experimental results show that CPS-merge has advantages over other methods even when κ_1 is incorrectly specified.

Next, we study the strategy for selecting m, the number of perturbed datasets, same as the number of perturbed clustering results. In **Main paper**, considering the load of computation, we set $m = 100$ for datasets HBMC and PBMC1 with sample sizes smaller than $100,000$, and $m = 50$ for PBMC2 with a sample size larger than $100,000$. Here, we evaluate the performance of CPS-merge across a range of m: $m = 50, 75, 90$ for HBMC and PBMC1, and $m = 25, 35, 45$ for PBMC2. As shown in Supplementary Fig D, the performance of CPS-merge increases with m. CPS-merge reaches the same level of performance as the results in Supplementary Table B when $m \geqslant 90$ for HBMC, $m \geqslant 75$ for PBMC1, and $m \geqslant 35$ for PBMC2. In summary, it is advantageous to use large m. We thus recommend to use a large m as permitted by the affordable computational load. Our empirical results show that $m \geqslant 90$ for datasets with sample sizes smaller than $100,000$ is adequate, while $m \geqslant 35$ for datasets with sample sizes over $100,000$ could yield competitive results.

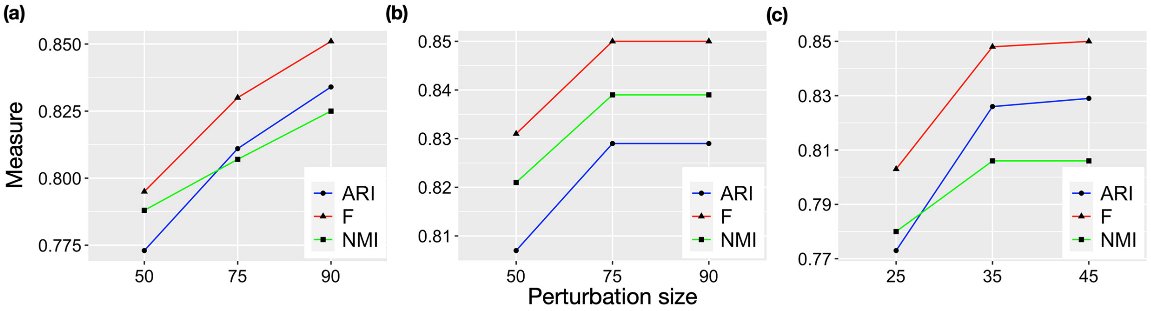

S1 Appendix Fig D. Sensitivity study on the performance of CPS-merge on three datasets. (a) HBMC. (b) PBMC1. (c) PBMC2.

References

[1] Fern XZ, Brodley CE. Solving cluster ensemble problems by bipartite graph partitioning. In: Proceedings of the twenty-first international conference on Machine learning; 2004. p. 36.
