# Supplementary material for: Multi-view clustering by CPS-merge analysis with application to multimodal single-cell data
Source: PLoS Comput Biol. 2023 Apr 17;19(4):e1011044. doi: 10.1371/journal.pcbi.1011044 (PMC10138214; doi:10.1371/journal.pcbi.1011044)
Supplement: Attachment — Submitted filename: ResponseLetter.pdf [file pcbi.1011044.s002.pdf]

Response Letter

Lixiang Zhang, Lin Lin, Jia Li

We thank the reviewers for their constructive suggestions. We have carefully addressed every review remark. Changes made in the manuscript in response to review comments are highlighted in red text. We provide below our itemized responses. The original review comments are copied here for clarity, each followed by our answers in blue text.

Reviewer 1

The topic is appropriate for publication, and the technical novelty of the paper is somewhat novel. Its contribution is moderately significant and the coverage of the problem is sufficiently comprehensive and balanced. The overall organization of the paper could be improved. The experimental results show that it obtains better performance than the state-of-the-art. However, following I have some minor questions that the authors should address to improve this work:

1. I want to advise authors to use GNN or some new methods rather than subspace learning. There are many similar methods. How to give novelty using new techniques or using the proposed methods on large-scale datasets?

   Answer: We have experimented with a popular DNN-based clustering method that works for multi-view data and provided new results. The network, namely Deep co-clustering (DeepCC), utilizes the deep autoencoder to learn a low-dimensional representation of the multi-view data, and employs a variant of Gaussian Mixture Model (GMM) for clustering. We follow the hyperparameter setting suggested by its author, and evaluate it on all 6 datasets (3 simulation datasets and 3 multimodal scRNA-seq datasets). According to the results (in main paper Table 2 and supplementary Table 2), DeepCC did not yield good clustering performance. Although the DNN-based method is able to handle large-scale datasts, it essentially tries to learn a shared low-dimensional representation of multiple views and thus ignores the complementary principle.

   Our proposed method is suitable for large-scale datasets. Because we do not need to pool data from multiple views, large-scale datasets can be analyzed separately in single views in relatively low dimensions (comparing with pooling). Moreover, the core parts of our method, i.e., aligning or merging clustering results, operate at the cluster level, while the number of clusters is usually much smaller than the number of data points. As answered for item 3 of Reviewer 2, the complexity of our method is expected to be linear in sample size. A detailed discussion is provided in an added paragraph on page 4. In our experiments, the PBMC2 dataset is of size $n = 161,764$. As shown in Table 2 in the manuscript, for this large dataset, our method achieves a greater performance gain comparing with the other smaller datasets.

2. There are many hyperparameters; how to tune them?

   Answer: We added a paragraph in the Results section (on page 13) on how to tune hyperparameters. A sensitivity analysis for the hyperparameters had been conducted with detailed results are provided in the Appendix/Supplementary.

3. How to use the algorithm in an online learning way? Or use neural networks to optimize such ideas?

   Answer: We added the following discussion on the extension to online learning in the last Section. "As suggested by one reviewer, exploring online learning for multi-view clustering is a promising direction for future research. Since CPS-merge analysis only uses cluster memberships but not the original data, it can be employed in an incremental learning mode as long as the clustering algorithms used in individual views allow online learning. Numerous clustering algorithms can be easily adapted to online learning, for instance, by representing previous data using per-cluster statistics, *e.g.*, mean vectors and covariance matrices. Based on these stored representations, new data batches can be clustered or assigned to new clusters without accessing past data. Neural

networks can also assist with online clustering. For instance, deep autoencoders can encode the original data in lower dimensions, which are typically easier to cluster, particularly under an online learning paradigm. Additionally, neural networks are frequently trained in batch mode, making them naturally suited for online learning. One challenge to consider for biomedical data, such as single-cell data, is that various data batches often contain batch effects that must be eliminated. Current methods for removing batch effects typically require processing all data in one view together, preventing effective online learning. Albeit interesting, how to overcome this issue in online learning is beyond the scope of our method here."

4. Somehow, it is incremental work; I still want to know the main difference and new.

   Answer: We thank the reviewer for pointing out the lack of clarity in our previous writing regarding the novelty or contribution of our work. We have revised the Introduction section to improve the organization and to better explain the novelty and contributions of our work. In particular, we added two paragraphs at the end of the Introduction section to describe the main contributions of the work and how it differs from existing methods.

5. Why the proposed method can obtain better performance than others? I would appreciate a broader discussion on why the proposed method performs better than the others.

   Answer: The main reason that our method can obtain better performance is that we exploit both the consensus principle and the complementary principle while existing late integration methods only consider the consensus principle. As explained in the introduction section with the added toy-data example, the relationship between multiple views is often complex and cannot be described by either principle alone. The consensus principle enables us to reduce noise in clustering by integrating multiple views. On the other hand, we analyze the Cartesian product clusters formed under the complementary principle. One major challenge of applying the complementary principle is that the number of Cartesian product clusters grows exponentially with the number of views. Many of these clusters exist because of randomness in data rather than the existence of meaningful subgroups. Our algorithm assesses the uncertainty level of each cluster and removes unstable clusters to overcome this difficulty.

6. I strongly recommend that authors release the source code along with the submission since the learning-based projects are typically open-source oriented to facilitate a fair assessment of the performance of the proposed methods for the community.

   Answer: We totally agree with the reviewer's suggestion. We have made the code publicly available along with implementation examples at the following site: https://github.com/LixiangZhang/CPS-merge.

7. Can you give toy-data figure to show the motivation clearer?

   Answer: We conducted simulation studies and provided detailed results (figures and tables) in the Appendix to better motivate our work and to show the potential advantage of considering both the consensus and complementary principle. Because the description of the simulations and the results is quite long, we cannot show the results in the main text. However, we agree that it is important to motivate our method using some toy datasets. In the added text, we explicitly point to those simulations and results in the introduction section (second to the last paragraph in this section).

Reviewer 2

1. This paper presents a late integration based multi-view clustering method for multi-modal single-cell data. In the Introduction, it should be better explained how the proposed method advances the late integration research and especially what limitations to the previous late integration based multi-view clustering methods have been tackled by the proposed method.

   Answer: We agree that how our method advances existing late integration methods should be better explained. First, we reorganized some paragraphs in the introduction section so that the

comparison with other late integration methods is more clear. Second, we added remarks about the limitations of the previous late integration methods and how we tackled these limitations. This review question is also closely related to the question raised by Reviewer 1 under item 4 and 5. We refer to the answers for item 4 and 5 of Reviewer 1 for more details.

2. In page 11, the contribution of each view to the clusters in the final result is evaluated. The weighting problem has been investigated in quite a few ensemble clustering methods, such as the local weighting strategy in Locally weighted ensemble clustering and Multidiversified ensemble clustering. Please explain whether the existing weighting strategies in ensemble clustering are feasible for the proposed work and what the advantages of the proposed weighting method are.

   Answer: We thank the reviewer for pointing out other well-designed methods used for evaluating the weights. In section "Evaluating cluster-wise contribution of each view", we proposed two methods to treat different scenarios. Our first method shares a core idea with existing methods, for instance, uncertainty implies importance/weight. We added references to existing methods on page 12 and discussed why those methods are not suitable for our case. One reason is that these methods assume that "the different partitions are independent", which does not hold for the usage scenarios we encounter here. Let us consider different partitions generated in a single view. Because they are created by the same clustering method on slightly perturbed data, the partitions are highly correlated. In the second usage scenario which we considered in the paper, because uncertainty is universally high across the views, the uncertainty-metric-based methods are inappropriate to apply. Thus we proposed the second method to handle this scenario.

3. The computational complexity of the proposed method should be analyzed. In recently, some large-scale ensemble clustering technique has been proposed, such as the ultra-scalable spectral clustering and ensemble clustering. It can be discussed whether the proposed framework can be extended to large-scale scenarios in the future work.

   Answer: We added a paragraph on page 4 to discuss the computational complexity of the proposed method. Because our algorithm only involves alignment of clusters but not the original data points, the complexity encountered in optimal transport is quadratic in the number of clusters, which is usually much smaller than the number of data points. As a result, if the data size increases, it only directly affects the generation of perturbed data (complexity is linear in data size) and the complexity of the clustering algorithms used in individual views. Many clustering algorithms in individual views have linear complexity in terms of data size. If the number of clusters does not change drastically, the complexity of our algorithm for integrating the clustering results will not increase much. We provide the discussion on computational complexity in a new paragraph at the end of page 4.

4. Some minor issues in the References: (i) For Ref. [36], it is suggested to use its journal version (https://doi.org/10.1109/TNNLS.2022.3192445) rather than its arXiv versions. (ii) For the discussions of the late integration methods in the third paragraph of the Introduction, it is strange that no references have been provided. Some late integration based multi-view clustering methods, such as [https://doi.org/10.1109/TKDE.2023.3236698], should be discussed specifically. In fact, the late integration methods merge the multiple partitions from multiple views, which resemble the ensemble clustering technique. Some discussions regarding the relationship between the late integration and the ensemble clustering are also suggested.

   Answer: We apologize for the confusion in the references. First, we corrected Ref. [36] as suggested. Second, we had several references for late integration methods, but they were provided before the third paragraph. That caused confusion. We have reorganized the paragraphs to avoid the confusion. In the updated manuscript, when we discuss in details the late integration methods, these references are cited again. Third, we added the new reference ([23]) suggested and discussed the work in a new paragraph at the end of page 2. We also commented on the relationship between late integration and ensemble clustering methods.
